# Implementation of the three good questions—A feasibility study in Dutch hospital departments

**DOI:** 10.1111/hex.12960

**Published:** 2019-09-27

**Authors:** Mirjam M. Garvelink, Marja Jillissen, Anouk Knops, Jan A. M. Kremer, Rosella P.M.G. Hermens, Marjan J. Meinders

**Affiliations:** ^1^ Radboud university medical center Radboud Institute for Health Sciences IQ Healthcare Nijmegen The Netherlands; ^2^ Centre De Recherche Sur Les Soins Et Les Services De Première Ligne De l'Université Laval (CERSSPL‐UL) Quebec QC Canada; ^3^ Department of Value Based Healthcare St Antonius Hospital Nieuwegein The Netherlands; ^4^ Radboud university medical center Improvement & Implementation Group Nijmegen The Netherlands; ^5^ Netherlands Patients Federation Utrecht The Netherlands

**Keywords:** feasibility study, patient communication, secondary care, shared decision making

## Abstract

**Objectives:**

To determine the feasibility of pragmatic implementation strategies for three good questions (in Dutch: Drie Goede Vragen; 3GV. What are my options; what are the risks and benefits related to these options; and what does this mean for my situation?) to increase shared decision‐making (SDM) efforts in Dutch secondary care, and identify barriers and facilitators of implementation.

**Methods:**

Convergent mixed‐method design: pre‐post surveys with patients attending one of six clinical departments in a Dutch Hospital, post‐intervention interviews with patients and health‐care professionals. Primary outcomes: feasibility (reach, use of 3GV). Secondary outcomes: SDM, experiences with 3GV and decision making. Interviews focused on barriers and facilitators of 3GV use. Interviews were content coded and categorized into determinants of behaviour change.

**Results:**

35% of the respondents who had heard of 3GV (52%) used all three questions. 3GV use did not lead to more SDM (SDMQ9 M = Δ0.3;SE = 2.2) but patients felt empowered to decide (88%) and to SDM (86%). Barriers were as follows: time investment, other SDM projects and perception that the need to use 3GV differs per patient/consultation. Respondents preferred to use 3GV as they saw fit for the consultation, instead of literally asking them. Facilitators: easy, accessible information materials that can be flexibly used.

**Conclusion:**

Implementation of 3GV seemed feasible, although influenced by contextual characteristics (eg type of decisions, patients, on‐going interventions). 3GV contributed to important elements of SDM, and respondents were willing to apply them in a way that suited their situation.

**Practice implications:**

We recommend continuation of current and new implementation strategies to enable 3GV implementation in secondary care.

## 1 BACKGROUND

Shared decision making (SDM) for health‐care decisions implies that patients, their informal caregivers and health‐care professionals share medical information and information about personal preferences, in order to make a value‐based and informed decision.^1^, ^2^, ^3^, ^4^ Several studies have shown the benefits of SDM with regard to reducing patients’ decisional regret and conflict, improving quality of life and leading to better treatment adherence and more conservative care.^5^ SDM is now a sine qua non of patient‐centred care, but it has not been widely implemented yet in health care.^6^, ^7^, ^8^, ^9^ Multiple barriers and facilitators have been found related to implementation of SDM.^6^, ^7^, ^8^, ^9^ Important barriers for health professionals included time constraints, lack of applicability due to patient characteristics and lack of applicability due to the clinical situation.^10^ Important facilitators included provider motivation to use SDM, its positive impact on the clinical process and its positive impact on patient outcomes.^10^ For patients, important barriers were related to a lack of knowledge and the power imbalance in the doctor–patient relationship.^7^ Several organizational characteristics have been identified as well as potential barriers, including organizational leadership, culture, resources, priorities, and teams and workflows.^9^

Numerous interventions exist to improve the adoption of SDM for specific treatment and screening decisions. These interventions focus on different aspects of SDM, as SDM may involve different steps that require different approaches to be achieved, particularly (a) determination of the decision point, people involved and their roles; (b) information exchange; (c) deliberation of values and preferences; (d) feasibility of options; (e) decision making; and (f) implementation and evaluation.^4^, ^11^, ^12^ Some interventions focus on deliberation and provide ways to clarify personal values in decision making, or how to communicate with health‐care professionals; others focus on the provision of disease‐specific information only, including treatment options, their risks/benefits, their likelihood of occurrence and patient values/preferences related to the risks/benefits. The sharing of such information is an important element of SDM.^1^, ^2^, ^13^ However, information about options, risks and consequences may change according to continuously evolving medical insights. Instead of continuously updating the information in decision support interventions, it may be easier to teach patients the right (generic) questions to obtain such information and teach health‐care professionals how to deal with answering these questions, and the SDM process that may follow. To this end, a generic tool has been developed consisting of three questions to ask your health‐care professional when facing any treatment decision: (a) what are my options?; (b) what are the risks and benefits related to these options?; and (c) what does this mean for my situation? 14, 15, 16, 17

Although no evidence exists that these three questions impact all aspects of SDM, some preliminary data on the benefits of the questions for certain components of SDM do exist. Moreover, the questions increase patients’ awareness about options and about their possible role in decision making about options on the patient's side, as well as greater information provision and behaviour supporting patient involvement on the health professionals’ side.^10^, ^11^, ^13^ Also, research has shown that both patients and health‐care professionals are generally positive about their use.^15^, ^16^, ^18^ However, additional SDM interventions (eg training, feedback) may be needed for health‐care professionals, to actually improve SDM during the consultation.^15^, ^16^

In 2015, the three questions as formulated in the MAGIC project^17^ have been translated to Dutch (known as ‘three good questions’/‘drie goede vragen’/‘3GV’), in a shared, consensus‐based initiative by the Dutch Federation of Patient Organizations (PFN) and the Dutch Federation for Medical Specialists (FMS), and in close collaboration with a translation bureau specialized in plain language translations, after which they were published on the PFN website for patients to find and use. In addition to this implicit mass media strategy, additional implementation strategies seemed necessary to actually achieve a positive impact on SDM. Therefore, to implement 3GV in secondary care, we adopted implementation strategies adapted to local contexts of six different hospital departments of the Radboud University Medical Center. This study aims to (a) determine the feasibility of implementation of 3GV in order to increase SDM efforts in Dutch secondary care and (b) to identify barriers and facilitators of implementation.

## 2 METHODS

### 2.1 Design

For this pilot study, we used a mixed‐method triangulation design, consisting of pre‐post surveys and post‐intervention interviews in five outpatient clinics (Departments) of the Radboud University Medical Center (Departments of Cardiology, Radiotherapy, Breast Cancer, Nephrology and Psychiatry) and one inpatient clinic (General Internal Medicine). The qualitative interviews were used to better understand the quantitative data as obtained from the surveys, using a convergent, sequential model in which qualitative and quantitative findings are analysed and interpreted separately and then combined to complement each other.^19^

### 2.2 Participants

Eligible participants were patients attending one of the six departments within one university medical centre, their health‐care professionals (physicians and nurses) and the project manager. Departments were selected based on their shown interest in implementing the 3GV. Patients had to be 18 years or older and able to sign informed consent. Each department defined their own target patient population (ranging from very specific groups of patients to every patient that visited the clinic) and which professionals participated. Hence, we invited (a) patients who visited the outpatient clinic (department of psychiatry), (b) patients who were admitted to the inpatient ward (department of general internal medicine) or (c) patients who visited the outpatient clinic for the first time (departments of cardiology, radiotherapy and nephrology). Patients with breast cancer were only invited if they were facing a treatment decision after being diagnosed. Different groups of patients completed the pre‐ and the post‐questionnaires and participated in the interviews.

### 2.3 Strategies to implement 3GV

Implementation strategies were as much as possible embedded in standard care and current improvement initiatives, which differed per department; hence, implementation strategies differed too.

#### 2.3.1 Implementation strategies for patients

**Figure 1:**
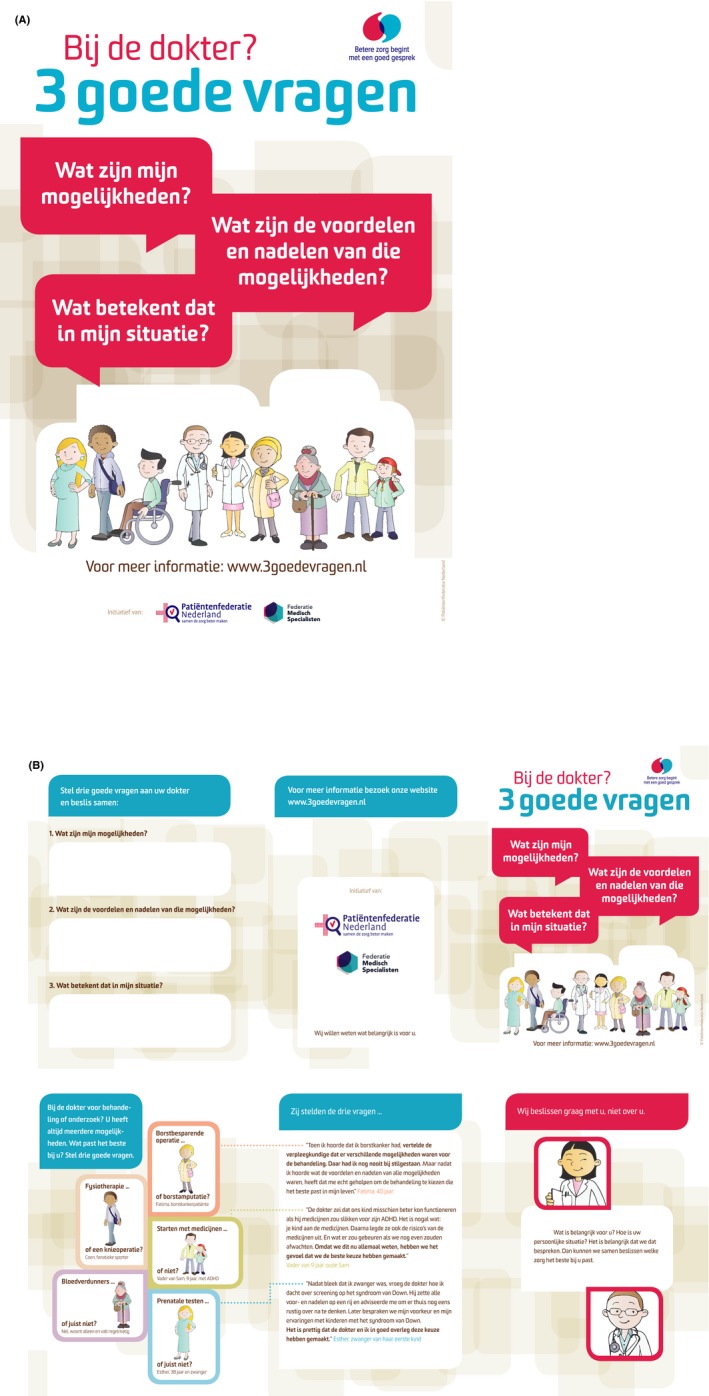
3GV poster (A) and 3GV brochure (B) (in Dutch)

1. Information brochures (Figure 1) were given by a nurse or resident during the consultation as part of an information package for all admitted patients or sent to the patient's (all patients) home before the consultation together with the confirmation letter for the appointment (Table 1).
2. Information was presented on digital screens and posters (Figure 1) in waiting rooms (all departments).
3. Pocket cards and posters were present in the consultation room (all departments).
4. Patient organizations (nephrology and PFN) published the materials on their websites.

**Table 1:** Implemetation strategies, number of completed surveys and conducted interviews, across the six participating hospital departments

Department	HCP Involved in implementation	Implementation strategies for professionals	Distribution of information brochures to patients	Type of patients involved	Survey		Interviews	
					Pre	Post	Patients	Professionals
Cardiology	2 cardiologists, 2 nurse specialists	1 + 4	sent home before consultation	Patients attending the outpatient clinic for the first time	32	42	2	2
General internal medicine	All nurses and residents	1 + 2 + 3 + 4+ organization of a theme month with attention for 3GV (for nurses and patients)	Handed out during the consultation	All clinical patients	53	32	2	2
Radiotherapy	All radiotherapists and residents	1	sent home before consultation	Patients attending the outpatient clinic for the first time	46	24	0	2
Breast cancer care	2 surgeons, 1 nurse specialist	1 + 2 + 3 + 4	sent home before consultation	Patients who have to make a treatment decision after diagnosis	14	6^a^	6	2
Nephrology	All residents	1 + 2 + 3 + 4	sent home before consultation	New patients, patients attending the outpatient clinic pre‐dialysis, transplant patients	18	29	1	2
Psychiatry	1 psychologist, 2 psychiatrists, 3 nurse specialists	1 + 2 + 3 + 4	sent home before consultation	Patients attending the outpatient clinic	31	5	1	2
Total					194	138	12	19

Note

Implementation strategies: (a) A general introduction meeting about SDM for all professionals (typically 30 min, during a regular staff meeting); (b) A 60‐min workshop to explain and train how to use the 3GV for health‐care professionals that were involved in the implementation (workshop 1); (c) An informative session to increase awareness of SDM in general and 3GV specifically and to learn from each other based on practice cases (workshop 2); (d) Shadowing or video‐taping consultations two consultations per health‐care professional in which the 3GV were used by an observer who had extensive experience in SDM training, physician‐patient communication and person‐centred care. The observer was present in the consultation room and used a structured rating list, based on the OPTION‐5. After each shadowing session, the professional received personal feedback. The observations were also used in workshop 2, to initiate group discussions between the professionals on their experiences, how to deal with particular situations and how to improve SDM behaviours.

Abbreviation: HCP, health‐care professional.

a Data from interviews used.

#### 2.3.2 Implementation strategies for professionals

For each department, a tailored implementation strategy was designed (Table 1), which could include:

1. A general introduction meeting about SDM for all professionals (typically 30 minutes, during a regular staff meeting);
2. A 60‐minute workshop to explain and train how to use the 3GV for health‐care professionals that were involved in the implementation (workshop 1);
3. An informative session to increase awareness of SDM in general and 3GV specifically and to learn from each other based on practice cases (workshop 2);
4. Shadowing or video‐taping two consultations per health‐care professional in which the 3GV were used by an observer who had extensive experience in SDM training, physician‐patient communication and person‐centred care. The observer was present in the consultation room and used a structured rating list, based on the OPTION‐5. After each shadowing session, the professional received personal feedback. The observations were also used in workshop 2, to initiate group discussions between the professionals on their experiences, how to deal with particular situations and how to improve SDM behaviours.

All strategies focused on practical aspects of implementation of 3GV and were easy to use in the actual practical context (hence ‘pragmatic’). The exact strategy differed per department (Table 1).

### 2.4 Primary and secondary outcomes

Our primary outcome was feasibility which was determined by qualitative and quantitative statements related to implementation of the 3GV, the percentage of patients who had heard of the 3GV (reach), and the extent to which 3GV were used in the consultation. Secondary outcomes were measures related to experiences with SDM, as measured with the SDMQ9,^20^ Collaborate^21^, ^22^, ^23^, ^24^ and control preference scale.^25^

### 2.5 Data collection methods and measurement instruments

Data collection took place between November 2014 and July 2015, and consisted of quantitative surveys and qualitative interviews.

- A Quantitative pre‐ and post‐implementation survey for patients captured socio‐demographic characteristics (sex, age, living situation), consultation characteristics (first or follow‐up appointment, consultation length, type of decision made), decision‐making process (SDMQ9,^20^ post only: CollaboRATE,^21^, ^22^, ^23^, ^24^ translated to Dutch (unpublished) according to the guidelines as provided by the developers of CollaboRATE^26^), the patient's perceived role in decision making (control preference scale 25), plus their use and experiences with 3GV. Data on the SDMQ9 and collaboRATE were recoded to calculate mean and total scores according to established methodologies:
  ͦ the SDMQ9 consists of 9 questions measured on a 5‐point Likert scale (Cronbach's α in our study was .96), which were recoded to an overall SDM score ranging from 0 to 100. A higher score indicates more SDM.^15^
  ͦ the CollaboRATE consists of three items measured on a 10‐point scale ranging from 0 to 9. We calculated top scores and average scores per item and for all three items together. To calculate CollaboRATE top scores, scores from 0 to 8 were interpreted as ‘absence of SDM’ whereas a ‘9’ was interpreted as ‘presence of SDM’. To calculate CollaboRATE mean scores, we calculated average scores.^16^, ^17^, ^18^, ^19^
- Qualitative, semi‐structured individual interviews with patients, health‐care professionals (physicians and nurses) and the project manager, post‐implementation, based on the model of behavioural change.^27^, ^28^ Key topics were as follows: to what extent were implementation strategies used and how were they evaluated? To what extent did the strategies contribute to implementation of 3GV? What factors influenced the use of 3GV? Finally, respondents were invited to provide feedback on 3GV materials and implementation strategies.

### 2.6 Sample size

We aimed for 25 completed surveys per department and per measurement moment in order to reach sufficient power for our measures of SDM (as per Ref.^21^, ^23^). In the breast cancer department, no post‐intervention questionnaires were handed out because during recruitment for the pre‐intervention questionnaires, it turned out to be too difficult to get patients with newly diagnosed breast cancer to complete a questionnaire due to the emotionally challenging period they were in. Instead, the most relevant items from the survey were included in the interview guide for this population.

### 2.7 Analysis

#### 2.7.1 Quantitative surveys

All data were entered in SPSS. We performed descriptive statistics on all data (calculation of n, %, means, SD, medians, ranges). No statistical tests were performed other than bivariate correlations between asking the 3GV, SDMQ9 and CollaboRATE. Due to fewer participants in 4 out of 6 departments, data were analysed and presented for all departments together, instead of per department. For the breast cancer department, we used data from interviews instead of post‐questionnaire results with regard to their experience with 3GV.

#### 2.7.2 Handling missing values

Missing values were coded as such and hence automatically excluded from analysis. To indicate the number of missing values per outcome, we provide the results per outcome together with the total number of responses per outcome. For calculation of SDMQ9 and CollaboRATE scores, we excluded cases where responses on one or more of the items were missing.

#### 2.7.3 Qualitative interviews

Interviews were audio‐recorded and transcribed verbatim. Any identifying information was removed from the interview transcripts. Two independent researchers performed a framework analysis^29^ starting with open content coding and subsequent thematic categorization of the codes into one of six determinants of behaviour change (ie beliefs and motivations, attitude, subjective norm, perceived behavioural control, intention, behaviour).^27^, ^29^ This enabled us to systematically assess the process of implementation, its barriers and facilitators. The analyses were supported by Atlas.ti software for qualitative analysis, to support coding and structuring the data. The quotes in the results section were translated from Dutch.

### 2.8 Ethics

The study protocol was submitted to the ‘Commissie Mensgebonden Onderzoek region Arnhem‐Nijmegen’ (2014‐1415). The committee determined that the *‘Wet Medisch‐Wetenschappelijk Onderzoek (WMO)*’ did not apply to this research; hence, we obtained a statement of ‘non‐objection’ from the ethics committee. Nevertheless, the research met all ethical regulations.

## 3 RESULTS

### 3.1 Response

Pre‐intervention, 455 patients were invited to participate, of which 194 patients completed a questionnaire. Post‐intervention, 319 patients were invited, of which 138 patients completed a questionnaire (Table 1). Response rates varied per department and ranged from 24% to 73%.

### 3.2 Quantitative results

#### 3.2.1 Characteristics of the survey participants and consultations

Participating patients were on average 58 years old, and sexes were equally distributed (Table 2). The majority of respondents (74%) lived together with a partner and/or with children.

**Table 2:** Survey respondent’ socio‐demographic and consultation characteristics

	Pre‐implementation (N = 194)		Post‐implementation (N = 138)	
	N (%)	Range per department	N (%)	Range per department
Sex, female	92 (47.4)	27.8%‐100%	74 (53.6)	20%‐100%
Age in years, M ± SD	57.6 ± 16.0	43.2 ‐ 65.0	59.3 ± 14.1	45.4 ‐ 62.3
Living situation, co‐habiting	143 (73.7)	9 ‐ 37	94 (72.3)	4 ‐ 21
First appointment: yes	85 (43.8)	30%‐93.5%	50 (36.2)	20%‐76.2%
Consultation length (minutes; M ± SD)	40.6 ± 32.2	22.0‐65.2	29.1 ± 19.4	18.7‐63.0
What decision was made				
Diagnostic testing	62 (32.0)	4‐21	57 (41.9)	1‐25
Follow‐up appointment	76 (39.6)	4‐21	52 (38.2)	1‐17
Referral to another professional	14 (7.3)	1‐6	12 (8.8)	0‐6
Start treatment	30 (15.5)	0‐13	33 (24.3)	0‐10
Stop treatment	5 (2.6)	0‐4	5 (3.7)	0‐3
Modify treatment	13 (6.7)	0‐5	7 (5.1)	0‐4
Something else	14 (7.2)	0‐7	16 (11.8)	0‐7

Most respondents visited their specialist for the first time, except for patients who visited the department of psychiatry (Table 2). The duration of the consultation varied from 20 minutes (Department of Cardiology) to 60 minutes (Department of Psychiatry). A variety of decisions were made during the consultations, of which the most frequently mentioned decisions were related to diagnostic testing or to follow‐up appointments.

#### 3.2.2 Feasibility

More than half of the participants prepared (any) questions before the consultation (pre: 65% and post: 57%), and more than 80% actually asked them (pre: 87% and post: 83%). Additionally, almost all participants were encouraged to ask questions (pre: 96% and post: 95%) and had the feeling their questions were adequately answered (pre: 93% and post: 89%).

**Figure 2:**
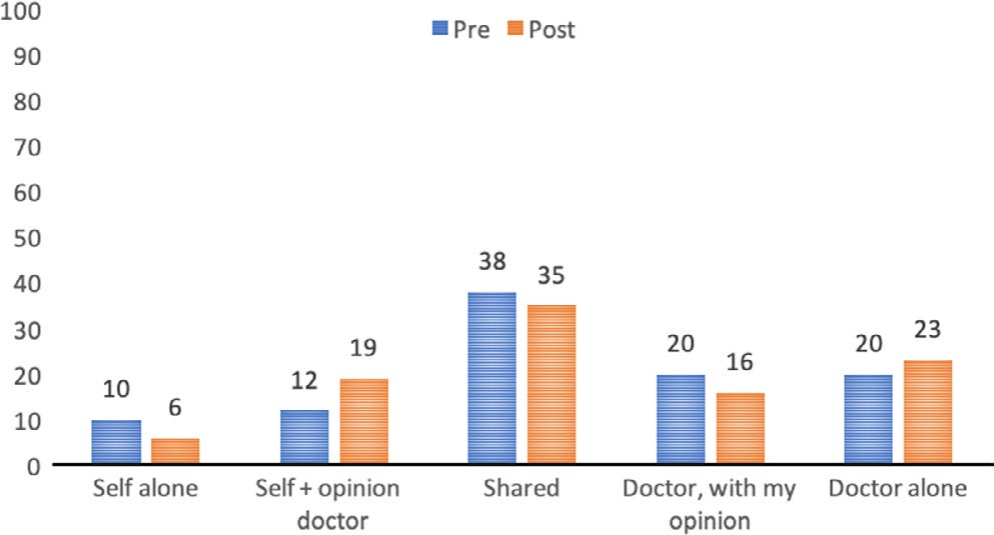
How participants used the 3GV during the consultation (based on questions about how patients used the 3GV: eg did you use the 3GV, and if so, how many questions did you ask?)

In the post‐questionnaire, about half of the participants (51.7% of 118 completed responses) indicated to have heard of the 3GV (reach). Of those, 35% had asked all three questions (Figure 2); 31% had prepared their appointment differently after learning about the 3GV. Respondents (n = 55) agreed that the 3GV helped them to ask questions (87%), get informed (86%), make a decision about next steps (78%), make a shared decision (86%), become aware of their options (62%) and feel allowed to participate in the discussion about treatment options (92%). Moreover, participants felt empowered to participate in decision making (88%), and 96% would recommend the 3GV to other people.

#### 3.2.3 Preliminary effects of 3GV on SDM during the consultations

**Figure 3:**
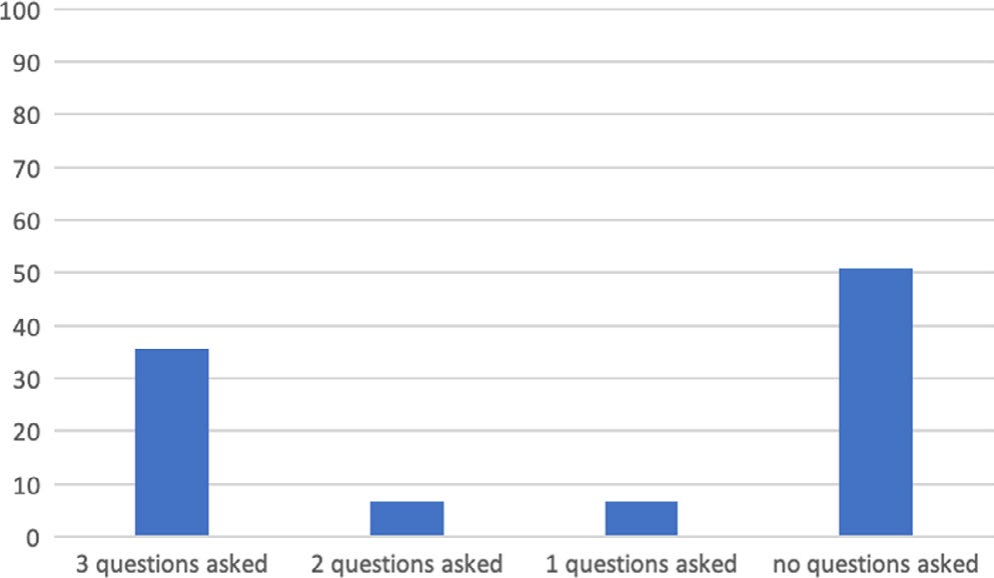
Perceived role in decision making as per the SDMQ9 (% pre‐ and post‐implementation)

Both pre‐ and post‐implementation measurements showed that about one‐third of the respondents perceived the decision‐making process to be shared, as appears from their perceived role (pre [N = 194]: 38%; post [N = 132]: 35%, Figure 3), the SDMQ9 (pre [N = 194] M = 75.4 ± 20.4; post [N = 138] M = 75.7 ± 19.8) and CollaboRATE (post [N = 132] M = 7.9 ± 1.3, top score 45.7%). However, there were no differences between pre‐ and post‐measures of SDM (not on single items either), nor were there correlations between asking the three questions and perceived SDM.

### 3.3 Qualitative results

#### 3.3.1 Barriers towards use and implementation of 3GV

##### Participants

Thirty‐one patients and health‐care professionals participated in the interviews, of which 12 patients, 10 physicians, 2 nurses and 7 project leaders. The majority of the participants (71%) were female (Table 3).

**Table 3:** Barriers and facilitators towards use and implementation of 3GV

	Barriers	Facilitators
Beliefs and motivations	Applicability depends on context (patient characteristics, decisions made)	There are different ways of using 3GV (literally ask and get information, to structure consultation)
	HCP pay enough attention to SDM already	3GV can make people realize that questions could be asked, and take away the threshold to do so
Attitudes towards the materials	Some questions are unclear or not considered useful	Positive attitudes to material as a whole: easy to understand, useful, and short but powerful
	Negative comments about illustrations	Layout found attractive
Perceived behavioural control	Decision point is too late in the assessed secondary care contexts	
	There is too much to discuss already	
Outpatient clinic vs inpatient ward	Outpatient clinic: None mentioned	Outpatient clinic: multiple decisions are made multiple types of questions can be asked patients see the same health‐care professional at each visit more time to prepare before the consultation more often accompanied by a loved one during the consultation
	On inpatient wards: unexpected decisions different professionals (depends on who is at shift). exact moment when the health‐care professional's visit takes place is often unexpected multiple health‐care professionals are present during the decision‐making moment (uncomfortable to ask many questions).	On inpatient wards: multiple decision moments and opportunities to use and practice with the 3GV nurses can help patients prepare to ask the 3GV to their physician.
Intention and behaviour (Use of 3GV)	Not always necessary to ask 3GV (health‐care professionals provide structured information and are open to respond to questions.)	3GV can be explicitly used in the consultation, or to structure the conversation
	Patients do not want more responsibility in deciding	
	Patients are too ill	
	Patients do not notice the 3GV materials because they are too pre‐occupied with their diagnosis or with the overload of other information they already receives.	
Suggestions for improvement of the implementation of 3GV	Lack of clear information about the objective of the project.	Informative meeting (ie workshop) clarified very well what was expected from the health‐care professionals with regard to the project.
	Distribution: when sent to patients as part of their appointment letter or the medical information package, some patients did not notice the materials between all other information	Timing of distribution allowed all patients to use them in preparation for their consultation
	Not all professionals were clear about their role in applying 3GV	Distribution spreading of information materials (sent home, available in waiting rooms/at the receptionist desk)
	Health‐care professional's role should be better clarified. This could be done by providing examples, for example by using videos	Make materials available at the hospital's information desk and other parts of the hospital, and use the internet to create awareness of 3GV
	To prepare for the informative meetings, more input from patients could be used to match the training to their perspectives	
	Video‐taping and shadowing professionals’ current behaviour are valuable educational methodologies	
	Informal caregivers could be better involved to prepare for consultations with 3GV	

##### Beliefs and motivations

Patients and health‐care professionals mentioned that patients already sufficiently asked all their questions which made the 3GV seem redundant. Health‐care professionals expected that the need and willingness to use 3GV may differ per patient, and per consultation, as not in all consultations decisions are being made.

That you realize why you do things, and I think I already do that, so for me it [the 3GV] was not really necessary, but I can imagine that there are many patients who do need them. But I can also imagine that not everybody is capable to think of good questions to ask. [patient about 3GV]

##### Attitudes towards the materials

Some respondents did not understand the question ‘what are my options’, as it was not specific enough. This question should be better clarified. Also, some participants thought that one of the illustrations (about breast cancer) was too confronting; other participants thought the illustrations were childish.

##### Perceived behavioural control

Due to the way in which specialized health care is organized, decisions were often made before patients had their appointment, so the information about 3GV came (too) late. Additionally, some health‐care professionals forgot about the 3GV in their consultation, because of the amount of information that had to be communicated during the consultation.

###### Organization of care: Outpatient clinics versus inpatients wards

On inpatients’ wards, the exact moment when the health‐care professional's visit takes place is often unexpected, and multiple health‐care professionals are present during decision making, which may make patients feel uncomfortable to ask many questions.

Also, decisions are made more unexpectedly during the health‐care professional's ‘round’, which makes it harder to prepare for decisions, and which makes it hard for loved ones to know when to be present for decision making.

#### 3.3.2 Intention and behaviour (Use of 3GV)

The most important reasons for patients not to use the 3GV were because they felt no need for it, as the health‐care professional already structured the information in a similar order as the questions or because enough opportunity was created to ask questions.

No, it was already clear from the structure that the doctor used in the conversation. She started discussing 5 treatments options and we weighed together how I thought about each option. [Patient who had not used the 3GV]

Other reasons for not asking the 3GV according to patients and health‐care professionals were that patients did not want more responsibility in deciding, that they were too ill, or too pre‐occupied with their diagnosis or with the overload of information they already received (so they did not notice the 3GV materials).

#### 3.3.3 Facilitators of use and implementation of 3GV

##### Beliefs and motivations

All participants believed the 3GV would lead to more patient involvement in medical decision making (Table 3). Health‐care professionals reported to use the questions to structure the consultations; patients thought that the questions could create awareness under patients that they are allowed to ask questions and to be involved in decision making.

Yes, and I also think it [3GV] will lower the threshold to do so. To ask those questions. As if they [patients] are like “hey, […] it is allowed. It is allowed to ask questions. [Patient]

##### Attitudes towards the materials

Most respondents were positive about the information materials. They thought that the 3GV were easy to understand, useful and simple but powerful. They thought the layout of the materials was attractive.

##### Perceived behavioural control

###### Organization of care: outpatient clinics versus inpatients wards

In outpatient clinics, patients have time to prepare themselves before the consultation and can bring a loved one to the consultation, who can support the use of 3GV. As the consultation time per patient in outpatient clinics is short, the 3GV can be used in preparation for the consultation which saves time in the consultation and increases efficiency. Additionally, in outpatient clinics many decisions are made, many types of questions can be asked, and patients often see the same health‐care professional at each visit.

On inpatient wards, patients may have more decision moments and more frequent opportunities to use (and practice with) the 3GV, compared with the outpatient clinic where an appointment has to be made first. Also, nurses can help patients prepare to ask their physician the 3GV.

##### Intention and behaviour (Use of 3GV)

Sometimes, the 3GV were explicitly used in the consultation, but more often they served to structure the conversation.

##### Suggestions for improvement of the implementation strategies for 3GV

Not all professionals were clear about their role in applying 3GV. The health‐care professional's role should thus be better clarified. This can be done by providing examples, for example in videos. Health‐care professionals mentioned that the educational meetings that were part of the implementation strategies would benefit from more patient input, to reflect their perspectives.

Video‐taping and shadowing professionals’ current behaviour were considered helpful, as it created awareness about how to improve patient involvement. These methods were seen as valuable educational methods to pursuit. Other educative methods that were considered useful to increase health‐care professional's awareness of SDM were reflective (mirror) conversations.

Definitely, and the feedback was really interesting […] because you never really receive this kind of feedback and we also do not know how colleagues do this, as it is all pretty individualistic. And yes, I thought it was good to amplify that, how we inform patients about their diagnosis and how we reason about the diagnosis. The way we […] work, sit behind that computer […] yes all very practical things. [physician about shadowing]

The moment that the 3GV materials were spread was positively evaluated by both patients and health‐care professionals. The moment allowed all patients to use 3GV in preparation of their consultation, either when materials were sent to their homes or when they were available in waiting rooms/at the receptionist desk. However, when 3GV brochures were sent to patient homes together with their appointment letter or the information package, some patients did not notice the 3GV materials between all other information. It was suggested to make the materials available at the hospital's information desk and other places in the hospital, as well as online.

Patients and health‐care professionals mentioned to seek for possibilities about how to improve the reach and actual use of 3GV. It was also mentioned that in supporting people to prepare for the consultation with 3GV, informal caregivers should be better involved.

So, I can recommend it [3GV] to all, without question. Even though some healthcare professionals will always have objections such as “as long as it does not bring more work” or “as long as I do not have to make more notes”. That is the overall concern that you hear everywhere when something new is being implemented. But if I was a patient, I would benefit from it, so I would say “yes”. [physician about 3GV]

## 4 DISCUSSION

In this project, we sought to determine the feasibility of pragmatic strategies to implement 3GV to increase shared decision‐making (SDM) efforts in Dutch secondary care. This also includes the identification of factors that act as barrier or facilitator in the implementation process. We found that implementation of 3GV created awareness for health professionals and patients about the possibility for patients to ask questions, but that only few patients used the 3GV, and that use of the 3GV did not lead to more SDM in the consultation as measured with validated SDM measures (SDMQ9, CollaboRATE). However, the majority of patients reported that the 3GV did help them to feel empowered to make a decision, get the feeling to be allowed to participate in the discussion about treatment options and make a shared decision. Health‐care professionals and patients appreciated the materials, but sometimes preferred to use them only to structure the consultations, instead of literally asking the 3 questions to get information. Moreover, health‐care professionals were not persuaded of the usefulness of 3GV for *all* patients, but they underline the importance of SDM and question‐asking/information sharing. The 3GV seem an easy and accessible method that may achieve this. The 3GV seemed especially fit for the outpatient clinic, less so for use on the inpatient ward. These results lead us to make three observations.

First, similar to other studies,^30^, ^31^ even after implementation, not all patients had heard of the 3GV, indicating that our implementation strategies could be improved. Moreover, the strategy used was considered a ‘minimal’ implementation strategy. Respondents made suggestions to improve implementation in the future. Elements in the implementation strategy that were considered essential by health‐care professionals were the educational part (workshops), including feedback (after video‐taping consultations) and shadowing. However, professionals mentioned that the objective of the project as a whole, as well as their role in it, was not clear, which may have hindered implementation. Although the 3GV are an intervention that should mainly be initiated by patients, professionals have an important role in creating the right consultation ambience to allow patients to do so.

Second, in line with other literature^32^, the availability of 3GV materials did not make all patients use the 3GV, partly because the availability of materials did not always lead to actual awareness of 3GV (many patients still had not heard of 3GV after implementation), but also because not all patients considered the questions necessary to get more information or a more active role in decision making. For example, it was often mentioned that health‐care professionals already provided structured information (thereby addressing the answers to the 3GV), which may be a result of the implementation strategies targeting the health‐care professionals. Additionally, decisions were often made in earlier consultations, so the questions came too late in the care process. Such organizational issues should be well thought‐through when implementing the 3GV.

Third, use of 3GV did not lead to more SDM as measured with validated tools. A likely explanation for this is that the 3GV focus primarily on information provision which is essential for decision making, and a prerequisite for other steps in SDM, but on its own it does not immediately lead to more SDM, as only few steps in the SDM process are addressed with these questions. Although we did not find differences on single items of the SDM‐Q‐9 either, which would indicate whether the physician paid more attention to specific steps of SDM, it is still possible that the biggest improvement after 3GV happened on a patient level. Indeed, one of the barriers for patients in SDM^7^ is the fear to ask questions, which is well addressed by the 3GV, because the 3GV create awareness about the possibility for patients to ask questions and provide example questions. Hence, 3GV may still be an important step to increase SDM use in clinical settings. Indeed, our findings show that 3GV helped patients to ask questions, to get informed and to be aware of their options. As well, 3GV made patients feel empowered to make a decision, get the feeling to be allowed to participate in the discussion about treatment options and make a shared decision. Indeed, post‐implementation patients reported somewhat more active roles in decision making too.

### 4.1 Limitations

Some limitations should be taken into account in interpretation of these results. First, although our total sample size was adequate, some departments were not able to include the required minimum of 25 respondents.^21^ Therefore, we merged all departments in the analyses. Merging all patients together to increase power meant that patients from six different hospital departments, with diverse medical problems and clinical characteristics, were put together for analysis. Future research should try to include more respondents to reach the necessary power per department and validate our findings per clinical department. Second, different groups of patients completed the pre‐ versus the post‐questionnaires. Although they had similar socio‐demographic and medical characteristics, it is possible that other (eg personal) characteristics influenced their role in decision making or evaluation of decision making. Third, one of our SDM measures (collaboRATE) was measured only post‐implementation because the Dutch translation was not ready at the time of the pre‐survey. Therefore, we were not able to assess how CollaboRATE scores actually changed after implementation of 3GV. We did have pre‐ and post‐data from the SDMQ9 and did not find pre‐post differences. However, at the latest International Shared Decision‐Making conference (July 7‐10, 2019, Quebec City, Quebec, Canada) some concerns were raised about the sensitivity of the SDMQ9 in picking up a patient's SDM experiences. Fourth, although the implementation strategies used were as much as possible adapted to and embedded in standard care and current improvement initiatives in order to facilitate implementation, this also meant that different implementation strategies were used and that we cannot determine which strategy is best. Fifth, the type of health‐care professionals involved in the implementation differed per department (eg physicians vs nurses), which may have influenced ease of implementation. Sixth, in this pilot, participating health‐care professionals and patients were from departments that were already interested in SDM, which may have facilitated implementation. Hence, the results of this paper may not be completely representative for other hospital departments with less motivated staff. Seventh, some differences in results reported by health‐care professionals versus patients indicate that health‐care professionals and patients have different perceptions of what entails a supportive conversation, information sharing and SDM. This has been found before^33^, ^34^ and should receive more attention in future research (eg by observing consultations in addition to the methods that were used in this pilot). Eighth, we assessed the number of questions asked by participants, but we have no data about which questions were omitted if not all 3GV were asked. This would be relevant to assess in future research, especially since we know from the interviews that participants had difficulty understanding the first question (what are my options).

## 5 CONCLUSION AND PRACTICE IMPLICATIONS

In conclusion, pragmatic implementation of 3GV seemed feasible in Dutch secondary care, although influenced by contextual characteristics of the situation (eg decisions to be made, type of patients, other on‐going interventions). Although this pilot project did not show a significant positive effect of 3GV on SDM in the consultations as measured with validated SDM instruments, both patients and health‐care professionals reported benefits of 3GV for question‐asking and decision making and were willing to apply them in a way that suited their (clinical) situation. Moreover, the 3GV contributed to important elements of SDM and have the potential to facilitate a full SDM process. Based on these conclusions, we recommend to continue current implementation strategies and look into adoption of new strategies that may reach an even larger public. Possibly, a more widespread, population‐based approach to implement 3GV in primary and secondary care would ensure that awareness is created on time for any decisions.

## CONFLICT OF INTEREST

This project was initiated by the Radboud University Medical Center and the Dutch Federation of Patients’ Organisations. All authors declare to have no conflicts of interest.

## Data Availability

The data that support the findings of this study are available from the corresponding author upon reasonable request.

## References

[1] Charles C , Gafni A , Whelan T . Shared decision‐making in the medical encounter: what does it mean? (or it takes at least two to tango). Soc Sci Med. 1997;44(5):681‐692. 9032835 10.1016/s0277-9536(96)00221-3

[2] Elwyn G , Edwards A , Kinnersley P . Shared decision‐making in primary care: the neglected second half of the consultation. Br J Gen Pract. 1999;49(443):477‐482. 10562751 PMC1313449

[3] Stiggelbout AM , Van der Weijden T , De Wit MP , et al. Shared decision making: really putting patients at the centre of healthcare. BMJ. 2012;344:e256. 22286508 10.1136/bmj.e256

[4] Legare F , Stacey D , Pouliot S , et al. Interprofessionalism and shared decision‐making in primary care: a stepwise approach towards a new model. J Interprof Care. 2011;25(1):18‐25. 20795835 10.3109/13561820.2010.490502 PMC3018136

[5] Shay LA , Lafata JE . Where is the evidence? A systematic review of shared decision making and patient outcomes. Med Decis Making. 2015;35(1):114‐131. 25351843 10.1177/0272989X14551638 PMC4270851

[6] Legare F , Elwyn G , Fishbein M , et al. Translating shared decision‐making into health care clinical practices: proof of concepts. Implement Sci. 2008;3:2. 18194521 10.1186/1748-5908-3-2 PMC2265300

[7] Joseph‐Williams N , Elwyn G , Edwards A . Knowledge is not power for patients: a systematic review and thematic synthesis of patient‐reported barriers and facilitators to shared decision making. Patient Educ Couns. 2014;94(3):291‐309. 24305642 10.1016/j.pec.2013.10.031

[8] Boland L , Graham ID , Legare F , et al. Barriers and facilitators of pediatric shared decision‐making: a systematic review. Implement Sci. 2019;14(1):7. 30658670 10.1186/s13012-018-0851-5 PMC6339273

[9] Scholl I , LaRussa A , Hahlweg P , Kobrin S , Elwyn G . Organizational‐ and system‐level characteristics that influence implementation of shared decision‐making and strategies to address them ‐ a scoping review. Implement Sci. 2018;13(1):40. 29523167 10.1186/s13012-018-0731-z PMC5845212

[10] Legare F , Ratte S , Gravel K , Graham ID . Barriers and facilitators to implementing shared decision‐making in clinical practice: update of a systematic review of health professionals' perceptions. Patient Educ Couns. 2008;73(3):526‐535. 18752915 10.1016/j.pec.2008.07.018

[11] Elwyn G , Frosch D , Thomson R , et al. Shared decision making: a model for clinical practice. J Gen Intern Med. 2012;27(10):1361‐1367. 22618581 10.1007/s11606-012-2077-6 PMC3445676

[12] Elwyn G , Durand MA , Song J , et al. A three‐talk model for shared decision making: multistage consultation process. BMJ. 2017;359:j4891. 29109079 10.1136/bmj.j4891 PMC5683042

[13] Stacey D , Legare F , Lewis K , et al. Decision aids for people facing health treatment or screening decisions. Cochrane Database Syst Rev. 2017;4:Cd001431. 28402085 10.1002/14651858.CD001431.pub5 PMC6478132

[14] McCaffery KJ , Morony S , Muscat DM , et al. Evaluation of an Australian health literacy training program for socially disadvantaged adults attending basic education classes: study protocol for a cluster randomised controlled trial. BMC Public Health. 2016;16:454. 27233237 10.1186/s12889-016-3034-9 PMC4884424

[15] Shepherd HL , Barratt A , Jones A , et al. Can consumers learn to ask three questions to improve shared decision making? A feasibility study of the ASK (AskShareKnow) Patient‐Clinician Communication Model((R)) intervention in a primary health‐care setting. Health Expect. 2016;19(5):1160‐1168. 26364752 10.1111/hex.12409 PMC5152736

[16] Shepherd HL , Barratt A , Trevena LJ , et al. Three questions that patients can ask to improve the quality of information physicians give about treatment options: a cross‐over trial. Patient Educ Couns. 2011;84(3):379‐385. 21831558 10.1016/j.pec.2011.07.022

[17] Lloyd A , Joseph‐Williams N , Edwards A , Rix A , Elwyn G . Patchy 'coherence': using normalization process theory to evaluate a multi‐faceted shared decision making implementation program (MAGIC). Implement Sci. 2013;8:102. 24006959 10.1186/1748-5908-8-102 PMC3848565

[18] Llewelyn H . Three questions a patient should ask. BMJ. 2017;358:j3988. 28947554 10.1136/bmj.j3988

[19] Creswell JW , Clark V . Choosing a mixed methods design In: Liechtenstein M, ed. Designing and Conducting Mixed Methods Research. Los Angeles: SAGE publications; 2007.

[20] Kriston L , Scholl I , Holzel L , Simon D , Loh A , Harter M . The 9‐item Shared Decision Making Questionnaire (SDM‐Q‐9). Development and psychometric properties in a primary care sample. Patient Educ Couns. 2010;80(1):94‐99. 19879711 10.1016/j.pec.2009.09.034

[21] Barr PJ , Forcino RC , Thompson R , et al. Evaluating CollaboRATE in a clinical setting: analysis of mode effects on scores, response rates and costs of data collection. BMJ Open. 2017;7(3):e014681. 10.1136/bmjopen-2016-014681 PMC5372080 28341691

[22] Barr PJ , Thompson R , Walsh T , Grande SW , Ozanne EM , Elwyn G . Correction: the psychometric properties of CollaboRATE: a fast and frugal patient‐reported measure of the shared decision‐making process. J Med Internet Res. 2015;17(2):e32. 25667387 10.2196/jmir.4272 PMC4353887

[23] Barr PJ , Thompson R , Walsh T , Grande SW , Ozanne EM , Elwyn G . The psychometric properties of CollaboRATE: a fast and frugal patient‐reported measure of the shared decision‐making process. J Med Internet Res. 2014;16(1):e2. 24389354 10.2196/jmir.3085 PMC3906697

[24] Elwyn G , Barr PJ , Grande SW , Thompson R , Walsh T , Ozanne EM . Developing CollaboRATE: a fast and frugal patient‐reported measure of shared decision making in clinical encounters. Patient Educ Couns. 2013;93(1):102‐107. 23768763 10.1016/j.pec.2013.05.009

[25] Degner LF , Sloan JA . Decision making during serious illness: what role do patients really want to play? J Clin Epidemiol. 1992;45(9):941‐950. 1432023 10.1016/0895-4356(92)90110-9

[26] Elwyn G .Translating collaborate. http://www.glynelwyn.com/translating-collaborate.html Accessed June 2019.

[27] Fishbein M , Yzer MC . Using theory to design effective health behavior interventions. Int Commun Assoc. 2003;13(2):164‐183.

[28] Godin G . Les Comportements Dans le Domaine de la Santé, Comprendre Pour Mieux Intervenir. Montreal, QC: Les presses de l'Université de Montreal; 2012.

[29] De Vries H , Mudde AN , Dijkstra A , Willemsen MC . Differential beliefs, perceived social influences, and self‐efficacy expectations among smokers in various motivational phases. Prev Med. 1998;27(5 Pt 1):681‐689. 9808799 10.1006/pmed.1998.0344

[30] Dutch Federation of Patients’ Organisations. [Report Three good questions] Rapport 3 goede vragen. Utrecht, the Netherlands, 2017.

[31] Nye A . Your health – your decision. evaluation & output report of the aqua workstream within the. National Shared Decision Making Programme. 2013; https://www.aquanw.nhs.uk/resources/shared-decision-making/Your-Health-Your-Decision-Evaluation-Report.pdf. Accessed on December 1, 2018.

[32] Garvelink MM , Lieshout JV , Palmen C , Maassen I , Faber M . Feasibility and effect of implementing the “Ask Share Know”‐questions in Dutch primary care. (submitted). 2018.

[33] Wunderlich T , Cooper G , Divine G , et al. Inconsistencies in patient perceptions and observer ratings of shared decision making: the case of colorectal cancer screening. Patient Educ Couns. 2010;80(3):358‐363. 20667678 10.1016/j.pec.2010.06.034 PMC2971658

[34] Hrisos S , Scott J , Vaittinen A , Thomson R . Illuminating Patient Push in the ‘Black Box’ of Shared Decision Making. Lyon, France: International Shared Decision Making conference (ISDM); 2017.

